# Psychometric evaluation of the Swedish version of the pure procrastination scale, the irrational procrastination scale, and the susceptibility to temptation scale in a clinical population

**DOI:** 10.1186/s40359-014-0054-z

**Published:** 2014-12-11

**Authors:** Alexander Rozental, Erik Forsell, Andreas Svensson, David Forsström, Gerhard Andersson, Per Carlbring

**Affiliations:** Division of Clinical Psychology, Department of Psychology, Stockholm University, Frescati Hagväg 8, SE-106 91 Stockholm, Sweden; Department of Behavioural Sciences and Learning, Linköping University, Linköping, Sweden; Department of Clinical Neuroscience, Division of Psychiatry, Karolinska Institutet, Stockholm, Sweden

**Keywords:** Procrastination, Psychometric evaluation, Irrational Procrastination Scale, Pure Procrastination Scale, Susceptibility to Temptation Scale

## Abstract

**Background:**

Procrastination is a prevalent self-regulatory failure associated with stress and anxiety, decreased well-being, and poorer performance in school as well as work. One-fifth of the adult population and half of the student population describe themselves as chronic and severe procrastinators. However, despite the fact that it can become a debilitating condition, valid and reliable self-report measures for assessing the occurrence and severity of procrastination are lacking, particularly for use in a clinical context. The current study explored the usefulness of the Swedish version of three Internet-administered self-report measures for evaluating procrastination; the Pure Procrastination Scale, the Irrational Procrastination Scale, and the Susceptibility to Temptation Scale, all having good psychometric properties in English.

**Methods:**

In total, 710 participants were recruited for a clinical trial of Internet-based cognitive behavior therapy for procrastination. All of the participants completed the scales as well as self-report measures of depression, anxiety, and quality of life. Principal Component Analysis was performed to assess the factor validity of the scales, and internal consistency and correlations between the scales were also determined. Intraclass Correlation Coefficient, Minimal Detectable Change, and Standard Error of Measurement were calculated for the Irrational Procrastination Scale.

**Results:**

The Swedish version of the scales have a similar factor structure as the English version, generated good internal consistencies, with Cronbach’s α ranging between .76 to .87, and were moderately to highly intercorrelated. The Irrational Procrastination Scale had an Intraclass Correlation Coefficient of .83, indicating excellent reliability. Furthermore, Standard Error of Measurement was 1.61, and Minimal Detectable Change was 4.47, suggesting that a change of almost five points on the scale is necessary to determine a reliable change in self-reported procrastination severity.

**Conclusions:**

The current study revealed that the Pure Procrastination Scale, the Irrational Procrastination Scale, and the Susceptibility to Temptation Scale are both valid and reliable from a psychometric perspective, and that they might be used for assessing the occurrence and severity of procrastination via the Internet.

**Trial registration:**

The current study is part of a clinical trial assessing the efficacy of Internet-based cognitive behavior therapy for procrastination, and was registered 04/22/2013 on ClinicalTrials.gov (NCT01842945).

## Background

Procrastination “to voluntarily delay an intended course of action despite expecting to be worse-off for the delay” (Steel, 2007, p. 66), is considered a prevalent self-regulatory failure that can result in personal distress and decreased well-being (Stead et al. 2010). In comparison to unintentionally postponing tasks and assignments, or having difficulties being self-assertive or prioritizing, procrastination is often regarded as a behavioral effect (Day et al. 2000), perpetuating most areas of life and causing troubles in the management of everyday commitments (Pychyl & Flett, 2012). Procrastination has been associated with stress and anxiety, fewer mental-health seeking behaviors, the development and exacerbation of physical disorders, as well as problems initiating and following through different wellness behaviors, e.g., rehabilitation, dental check-ups, and physical exercise (Sirios, 2004; 2007). In addition, procrastination is also associated with poorer performance in school as well as work, and can have a detrimental effect on both financial decisions and career development (Steel et al. 2001; Tice & Baumeister, 1997; O’Donoghue & Rabin, 1999). Hence, procrastination can become severely debilitating, leading to major psychological suffering and have a negative impact on quality of life (Sirios et al. 2003).

Procrastination is estimated to affect approximately one-fifth of the adult population and half of the student population (Day et al. 2000; Harriott and Ferrari 1996). Studies also suggest that the number of people experiencing difficulties due to procrastination is on the rise, presumably because of greater access to immediate gratification through the use of modern information technology (Steel, 2012). However, the nature of procrastination is still unclear, and various ways of defining, conceptualizing, and explaining procrastination have been proposed (Steel, 2007). For instance, in an attempt to explore the relationship between procrastination and heredity, Gustavsson et al. (in press) found a genetic link between procrastination and impulsivity. Prior research has also investigated the association between different personality factors and procrastination, indicating that, in particular, a high degree of impulsiveness and a lack of self-control seems to be involved (Specter & Ferrari, 2000; Tice & Baumeister, 1997). Different theories of procrastination have also been put forward using motivational concepts, most recently the Temporal Motivational Theory (Steel & König, 2006). According to Steel (2007), engagement in a given course of action is related to the expectation of achieving an anticipated outcome, the value of that outcome, the timing of the outcome, and the sensitivity to delay. Procrastination is thus characterized by having the intention to initiate or complete a task or assignment that will generate a certain value in the long run, but instead finding oneself pursuing other activities that are more readily enjoyable because of the timing of the reward and the ability to postpone gratification (Steel, 2010).

Different self-report measures have been developed in order to assess the occurrence and severity of procrastination, as well as to test the conceptual underpinnings of different theories of procrastination. Mann (1982; 1997) presented the Decisional Procrastination Questionnaire consisting of 30 items based on the notion of decisional procrastination, e.g., “I feel as if I’m under tremendous time pressure when making decisions” (item 1). Lay (1986) on the other hand developed the General Procrastination Scale, comprising 20 items of dilatory tendencies, e.g., “I generally delay before starting on work I have to do” (item 9). Furthermore, McCown et al. (1989) introduced the Adult Inventory of Procrastination, another general measure of procrastination, which includes fifteen items, e.g., “I don’t get things done on time” (item 5). Solomon and Rothblum (1984) put forward the Procrastination Assessment Scale for Students, measuring the level of procrastination in six domains of curricular activity and the reasons behind procrastination, e.g., “You were concerned the professor wouldn’t like your work” (item 19). However, according to a review by Steel (2010), the theoretical basis of many of the self-report measures have been found to be incoherent, particularly in terms of the idea of being able to differentiate various types of procrastination, i.e., arousal, avoidant, and decisional. It has also been suggested that decisional procrastination involve decisional avoidance rather than procrastination per se (Steel, 2007). In addition, Steel (2010) evaluated the psychometric properties of the self-report measures using factor analysis, revealing three distinct factors: a broad factor consisting of more general procrastination items, a second factor characterized by items that relate to keeping appointments and being in a rush, and a third factor that included items of promptness and the ability to perform tasks and assignments immediately. However, Steel (2010) argued that the results provided little evidence for a three-dimensional construct, particularly as only the first factor seemed to fit the definition of procrastination as being a voluntary delay. Steel (2010) therefore developed a new self-report measure using only items with the highest loadings on the first factor in the factor analysis, the Pure Procrastination Scale (PPS), consisting of twelve items deemed to determine dysfunctional delay, which was tested over the Internet on an English-speaking non-clinical population of 4169 participants. Furthermore, Steel (2010) proposed two additional self-report measures, the Irrational Procrastination Scale (IPS), which can be used as a parallel form to assess procrastination and allowing them to share validation efforts, and the Susceptibility to Temptation Scale (STS), examining the sensitivity to distractions and immediate gratification.

The results from Steel (2010) suggest that a single latent variable is sufficient to explain the nature of procrastination, namely, dysfunctional delay, and that self-report measures trying to distinguish different types of procrastination are unwarranted. Similar results were obtained by Rebetez et al. (2014) when evaluating a French version of the PPS on a French-speaking non-clinical population of 245 participants. Hence, the PPS may become a valuable instrument for determining the occurrence and severity of procrastination, particularly in a clinical context where valid and reliable self-report measures are important in differential diagnosis and the assessment of treatment outcome. However, although Steel (2010) and Rebetez et al. (2014) have provided preliminary evidence for the use of the PPS, no attempt has yet been made to implement it in a clinical population. The current study thus seeks to explore the psychometric properties of the PPS in a clinical trial of Internet-based cognitive behavior therapy for procrastination (Rozental & Carlbring, 2013). Furthermore, both the IPS and the STS are included in the analysis, as well as self-report measures of depression, anxiety, and quality of life, to further investigate the relationship between the respective instruments, as well as the potential association between self-report measures of procrastination and other types of psychiatric disorders. The purpose of the current study is thus to 1) explore the factor structure of the PPS, the IPS, and the STS in a self-referred clinical population 2) examine the discriminant and construct validity and reliability of the PPS, the IPS, and the STS in order to assess their psychometric properties, 3) evaluate the usefulness of the IPS as a self-report measure administered in a clinical trial by determining its test-retest reliability and minimal detectable change, and 4) investigate the correlations between the PPS, the IPS, and the STS, and self-report measures of depression, anxiety, and quality of life.

## Methods

### Participants

The current study is part of a clinical trial assessing the efficacy of Internet-based cognitive behavior therapy for procrastination (Rozental & Carlbring, 2013), and was registered 04/22/2013 as a clinical trial on ClinicalTrials.gov (NCT01842945). Participants were recruited through reports in the Swedish media, advertisements on the Internet, and through information on Facebook (Ramo et al., 2014). Eligibility for treatment was determined via an online screening process consisting of open-ended questions and several self-report measures of procrastination, depression, anxiety, and quality of life.

**Figure 1:**
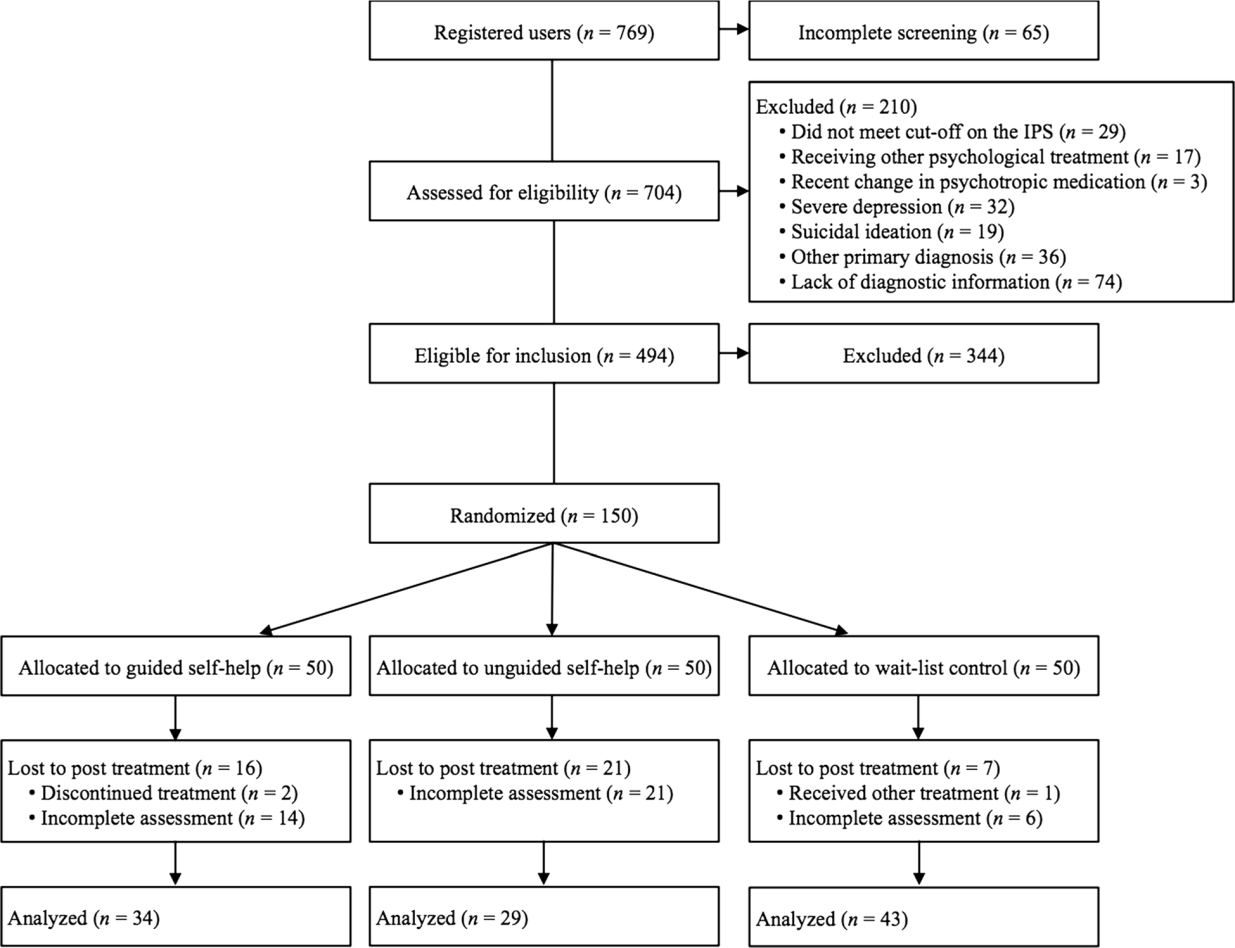
**Flow chart of participants throughout the current study.** IPS = Irrational Procrastination Scale.

In total, 710 participants completed the online screening process. Missing information on the sociodemographic characteristics were, however, found for six participants, but these were nonetheless included in the subsequent analyses as they provided complete values on the self-report measures. In the clinical trial (Rozental & Carlbring, 2013), 494 participants were eligible for inclusion based on the following criteria: Swedish residency, fluent in Swedish, at least 18 years old, having access to a computer with Internet, and suffering from problems primarily related to procrastination based on a minimum of 32 points on the IPS (Steel, 2012). Exclusion criteria were: ongoing psychological treatment, psychotropic medication unless the dosage had been stable for at least twelve weeks prior to entering treatment, and other psychiatric conditions regarded as better cared for elsewhere, e.g., bipolar disorder, schizophrenia, psychosis, ADHD/ADD, and severe misuse of alcohol or drugs. In addition, severe depression and suicidal ideation were also reasons for exclusion, as indicated by having a minimum of 32 points, or scoring 3 points or above on question 9 regarding suicidality on the Montgomery Åsberg Depression Rating Scale – Self-report version (MADRS-S; Svanborg & Åsberg, 2001). For a full presentation of inclusion and exclusion criteria, consult the study protocol by Rozental and Carlbring (2013). A complete flow chart of the clinical trial can be seen in Figure 1. Enrollment and randomization into three conditions, guided self-help, unguided self-help, and wait-list control, however, involved 150 participants, as this was the maximum number of participants that were to be included in the clinical trial. Of these, only the 50 participants in the wait-list control were used in the investigation of test-retest reliability in the current study. Hence, this is the only analysis in the current study that is affected by the cut-offs and exclusion criteria of the clinical trial by Rozental and Carlbring (2013). With this exception, when assessing the psychometric properties of the self-report measures in the current study, all 710 participants were included, regardless of their baseline severity level of procrastination, or if they were excluded from the clinical trial due to fulfilling one or more exclusion criteria. The sociodemographic characteristics of all participants can be found in Table 1.

**Table 1:** **Sociodemographic characteristics of the participants**

	**Screening sample**	**Wait-list sample**
	**(** ***n*** **= 710)**	**(** ***n*** **= 50)**
Gender: *n* (% female)	308 (43.4)	23 (46.0)
Age (years): *M* (*SD*)	38.59 (11.0)	41.56 (9.9)
**Marital status:** ***n*** **(%)**		
Single	208 (28.7)	17 (34.0)
Married/Partner	448 (63.1)	28 (56.0)
Divorced/Widow	42 (5.9)	4 (8.0)
Other	10 (1.4)	1 (2.0)
Children: *n* (% yes)	340 (47.9)	22 (44.0)
**Highest educational level:** ***n*** **(%)**		
Middle school	18 (2.5)	1 (2.0)
High school/college	287 (40.4)	13 (26.0)
University	372 (52.4)	32 (64.0)
Postgraduate	27 (3.8)	4 (8.0)
Sick leave: *n* (%)	17 (2.4)	0 (0.0)
Previous psychological treatment: *n* (% yes)	322 (45.4)	24 (48.0)
Previous psychotropic medication: *n* (% yes)	199 (28.0)	11 (22.0)

*Note:* Screening sample contains missing values on the sociodemographic characteristics of six participants and is therefore based on *n* = 704. However, all subsequent analyses include data from *n* = 710.

### Procedure

Participants were required to log on to a secure online interface requiring registration and electronic identification, i.e., SLL Certificates, in order to complete an automated and fully computerized online screening process, minimizing the risk of data loss or data distortion (Carlbring et al., 2007; Thorndike et al., 2009). All data were stored encrypted in adherence with the Swedish Personal Data Act (Datainspektionen, 1998). When completing the online screening process and registering for the clinical trial, participants received an auto generated identification code, e.g., 1234abcd, ensuring anonymity throughout the screening process, treatment period, and analysis of the results. For the wait-list control, self-report measures using the IPS were administered weekly throughout the waiting period using the secure online interface, and only reminders to complete the self-report measures were sent to the participants’ private email. All participants completed the screening process during the period of August through September 2013, and the weekly measures were carried out during the treatment period, ranging from September to November 2013.

## Ethics

The clinical trial, which the current study is a part of, received ethical approval from the Regional Ethical Board in Stockholm, Sweden (Dnr 2013/974-3175). Written informed consent was required by all participants in order to be considered eligible for participation. Great consideration was given to ensure that no participants were included while having another condition that might have required more immediate attention, in which case they were contacted with information on where to find relevant help. In addition, deterioration was closely monitored by the study supervisors in case the condition of a participant worsened and might require more specialized care. Potential negative effects were also investigated using open-ended questions concerning their characteristics and severity at post treatment assessment (Rozental et al., 2014), and reliable deterioration was explored using the Reliable Change Index (Boettcher et al. 2014). For ethical reasons, the participants in the wait-list control received unguided self-help after the first treatment period had ended.

### Measures

Included in the current study were two self-report measures of procrastination, the PPS (Steel, 2010), and the IPS (Steel, 2010), as well as one self-report measure of susceptibility to temptation, the STS (Steel, 2010), all of which were developed and tested over the Internet. The instruments were translated into Swedish by an authorized translator (see the Table 2 for both the English and Swedish versions). The PPS features twelve items measuring the prevalence of procrastination and was developed to increase the validity of several already existing procrastination scales (Steel, 2010). The English version of the PPS has a good internal consistency, Cronbach’s α = .92, and shows convergent validity with other related measures. The IPS features nine items measuring the degree of irrational delay causing procrastination, and its English version has yielded a good internal consistency, Cronbach’s α = .91, and correlates with PPS at *r* = .87, or *r* = .96, after correcting for attenuation due to unreliability (Steel, 2010). The STS features eleven items measuring the susceptibility to temptation, affecting the ability to initiate and complete tasks and assignments. The English version of the STS has a good internal consistency, Cronbach’s α = .89, and correlates with both the PPS and the IPS at *r* = .69 (Steel, 2010).

**Table 2:** **Original and translated versions of the self-report measures**

**Pure Procrastination Scale (PPS), with the Swedish version given in italics**		
	English version	Swedish translation
PPS1	I delay making decisions until it’s too late	*Jag skjuter upp beslut tills det är försent*
PPS2	Even after I make a decision I delay acting upon it	*Även efter att jag har fattat ett beslut dröjer det innan jag agerar i enlighet med det*
PPS3	I waste a lot of time on trivial matters before getting to the final decisions	*Jag kastar bort mycket tid på bagateller innan jag fattar ett slutgiltigt beslut*
PPS4	In preparation for some deadlines, I often waste time by doing other things	*När jag måste hålla en tidsgräns slösar jag ofta bort tiden på annat*
PPS5	Even jobs that require little else except sitting down and doing them, I find that they seldom get done for days	*Även när det gäller arbeten som inte är särskilt krävande kan det ta mig flera dagar att slutföra dem*
PPS6	I often find myself performing tasks that I had intended to do days before	*Jag ägnar mig ofta åt saker som jag hade tänkt att göra för flera dagar sedan*
PPS7	I am continually saying “I’ll do it tomorrow”	*Jag säger hela tiden att “det där gör jag imorgon”*
PPS8	I generally delay before starting on work I have to do	*Jag väntar vanligtvis med att påbörja ett arbete som jag måste göra*
PPS9	I find myself running out of time	*Det känns som om tiden inte räcker till*
PPS10	I don’t get things done on time	*Jag får inte saker och ting gjorda i tid*
PPS11	I am not very good at meeting deadlines	*Jag är inte bra på att hålla utlovade tider*
PPS12	Putting things off till the last minute has cost me money in the past	*Att skjuta upp saker och ting till sista minuten har tidigare stått mig dyrt*
**Irrational Procrastination Scale (IPS), with the Swedish version given in italics**		
	English version	Swedish translation
IPS1	I put things off so long that my well-being or efficiency unnecessarily suffers	*Jag skjuter upp saker och ting så pass länge att mitt välbefinnande eller min effektivitet blir lidande*
IPS2	If there is something I should do, I get to it before attending to lesser tasks (R)	*Om det är något jag borde göra, tar jag tag i det innan jag börjar med mindre betydelsefulla uppgifter (R)*
IPS3	My life would be better if I did some activities or tasks earlier	*Jag skulle må bättre om jag slutförde saker och ting tidigare*
IPS4	When I should be doing one thing, I will do another	*När jag borde göra en sak så gör jag något annat istället*
IPS5	At the end of the day, I know I could have spent the time better	*När dagen är slut upplever jag att jag hade kunnat utnyttja min tid bättre*
IPS6	I spend my time wisely (R)	*Jag använder min tid väl (R)*
IPS7	I delay tasks beyond what is reasonable	*Jag skjuter upp mina uppgifter mer än vad som är rimligt*
IPS8	I procrastinate	*Jag förhalar saker och ting*
IPS9	I do everything when I believe it needs to be done (R)	*Jag gör allt när jag anser att det behöver göras (R)*
*Note:* Items 2,6, and 9 are scored in reverse (R)		
**Susceptibility to Temptation Scale (STS), with the Swedish version given in italics**		
	English version	Swedish translation
STS1	I will crave a pleasurable diversion so sharply that I find it increasingly hard to stay on track	*Jag har ett så stort behov att ägna mig åt annat som är angenämt att jag får allt svårare att koncentrera mig på det jag ska göra*
STS2	I feel irresistibly drawn to anything interesting, entertaining, or enjoyable	*Jag känner en oemotståndlig dragningskraft till allt som är intressant, underhållande eller trevligt*
STS3	I have a hard time postponing pleasurable opportunities as the gradually crop up	*Jag har svårt att skjuta upp nöjen i samband med att de dyker upp*
STS4	When an attractive diversion comes my way, I am easily swayed	*Jag blir lätt distraherad när det dyker upp något som lockar*
STS5	My actions and words satisfy my short-term pleasures rather than my long-term goals	*Det jag säger och gör skänker mig en kortsiktig njutning snarare än att tillgodose mina långsiktiga mål*
STS6	I get into jams because I will get entranced by some temporarily delightful activity	*Jag får problem eftersom jag lätt blir distraherad av en tillfällig och tilltalande aktivitet*
STS7	It takes a lot for me to delay gratification	*Det krävs en stor uppoffring för mig att skjuta upp något som ger mig tillfredsställelse*
STS8	When a task is tedious, again and again I find myself pleasantly daydreaming rather than focusing	*När jag jobbar med en tråkig uppgift händer det ofta att jag dagdrömmer om annat än att fokusera på mitt arbete*
STS9	When a temptations is right before me, the craving can be intense	*Om jag står inför något som frestar mig så upplever jag ett starkt begär att falla till föga*
STS10	I choose smaller but more immediate pleasures over those larger but more delayed	*Jag väljer mindre men mer omgående nöjen än de som är större och tar längre tid att nå*
STS11	I take on new tasks that seem fun at first without thinking through the repercussions	*Jag tar på mig nya uppgifter som framstår som roliga utan att tänka igenom vilka följder det kan få*

Additional self-report measures of depression, anxiety, and quality of life were also included in the current study, using the Swedish version of the MADRS-S (Svanborg & Åsberg, 2001; Holländare et al. 2010), the Generalized Anxiety Disorder Assessment 7-item (GAD-7; Spitzer et al., 2006; Dear et al., 2011), and the Quality of Life Inventory (QOLI; Frisch et al., 1992; Lindner et al., 2013). The MADRS-S is a self-assessment version of the MADRS, featuring nine items measuring changes in mood, anxiety, sleeping patterns, appetite, concentration, initiative, emotional engagement, pessimism and attitude towards life. The MADRS-S has been evaluated over the Internet with an internal consistency similar to the paper version, Cronbach’s α between .73 and .81, as well as a high correlation between the formats, *r* = .84 (Holländare et al. 2010). The GAD-7 features seven items for assessing anxiety and screening for generalized anxiety disorder, and has been assessed over the Internet with a good internal consistency, Cronbach’s α = .79, and with large correlations to other related measures of anxiety and worry at post treatment, *r* = .68 to .76 (Dear et al., 2011). The QOLI features 32 items concerning 16 areas of life rated by the participants with regard to importance and satisfaction, and has been shown to have a good internal consistency, Cronbach’s α between .71 and .83, when administered over the Internet (Lindner et al., 2013).

### Statistical analysis

Prior to analysis, the distribution of data was assessed and levels of skewness and kurtosis were found to be acceptable for analysis. In addition, Keiser-Meyer-Olkin’s test of sampling adequacy (KMO) and Bartlett’s test of sphericity showed that the data was highly suitable for factor analysis.

Principal Component Analysis (PCA) with Varimax-rotation was performed to assess the component structure of the Swedish versions of the IPS, the PPS, and the STS in order to explore how these instruments behave in a novel sample as well as language, rather than confirming any hypothesis regarding their respective component structure. This approach was chosen based on the recommendations by Cichetti (1994), as the current sample had different characteristics than the ones used in the studies by Steel (2010) and Rebetez et al. (2014). Included in the current study are participants who perceived themselves to be in need of treatment for procrastination, while the samples in the original studies by Steel (2010) and Rebetez et al. (2014) were not explicitly seeking treatment. Furthermore, as the current study intended to investigate the component structure of the instruments in Swedish, the approach was explorative, and the use PCA was thus deemed appropriate.

Analyses of how all of the different measures correlated with each other were also performed, including the MADRS-S, the GAD-7, and the QOLI. Corrections for attenuation due to unreliability was performed using the formula *r*_*x’y’*_=*r*^*xy*^ / *(*√*r*_*xx*_*r*_*yy*_*)* (Zimmerman, 2007).

Cronbach’s α was used as an indication of internal consistency. A two-way random effect single measure was used as an indicator of Intraclass Coefficient Correlation (ICC; Baldwin et al., 2011), following the recommendations in McGraw and Wong (1996). Absolute agreement, in accordance to McGraw and Wong (1996), was used as a measure of coherence.

Standard Error of Measurement (SEM) was defined as the square root of the mean square error in the ANOVA (Weir 2005). This was then used to calculate the Minimal Detectable Change (MDC) defined as SEM x 1.96 x *k,* where 1.96 represents a 95% confidence interval in a *z*-distribution, and *k* is the number of measurements (in our case 2) as described by Wier (2005). All statistical analyses were made in SPSS version 21.

## Results

### Distribution of data

An initial analysis of the data showed that IPS, PPS and STS were approximately normally distributed, see Table 3. There were some high values in the sample, but this was probably due to the fact that the participants were seeking treatment for difficulties related to procrastination. According to the results of the KMO and Bartlett’s test of sphericity the data was suitable for a factor analysis.

**Table 3:** **Data distribution for the Irrational Procrastination Scale (IPS), the Pure Procrastination Scale (PPS), and the Susceptibility to Temptation Scale (STS)**

	**Mean (SD)**	**Skewness**	**Kurtosis**	**KMO**	**Bartlett**
**IPS**	38.47 (3.62)	-0.496	0.070	0.844	*p <* 0.0001
**PPS**	49.26 (5.69)	-0.402	-0.231	0.831	*p <* 0.0001
**STS**	42.02 (7.07)	-0.428	-0.017	0.909	*p <* 0.0001

*Note:* Table of mean, skewness, kurtosis, KMO test of sampling adequacy and Bartlett’s test of sphericity.

### Validity

#### Factor validity of the IPS

The PCA with Varimax-rotation of the IPS generated two factors with eigenvalues of one or more. The scree-plot was examined to ensure that the two factor solution seemed reasonable, after which it was retained. For the exploratory purposes of this analysis the cut off for factor loadings was set to .40. Smaller coefficients are not reported in Tables 3 and 4. The first factor reflected the suggestion by Steel (2010) regarding the unidimensionality of the IPS, which seems to measure general procrastination. It accounted for 35% of the variance and had an eigenvalue of 3.24. The second factor extracted had an eigenvalue of 1.06 and contained items 2, 6 and 9, which are scored in reverse, for instance, “I do everything when I believe it needs to be done” (item 9). Factor loadings for the items in the IPS are presented in Table 4. The second factor is however most likely an artifact of the instrument, reflecting the fact that the participants simply missed the double negatives or reversed items despite being included in the scale to prevent mindless responses. Artifactual response factors containing all reversed items in a scale are a relatively common issue in scale development (Hinkin, 1995). This seems reasonable since the item-scores of 1 and 2 points, that is, after the scores have been reversed, appeared in the current sample 129 times in total for the IPS, with 105 of these being responses to the three reversed items. Furthermore, “I spend my time wisely” (item 6), cross-loaded and had a lower loading on the second factor, possibly reflecting the relatively short and concise phrasing of the item, leaving it less open to misinterpretation than items 2 and 9.

**Table 4:** **Rotated component matrix for a two factor solution of the Irrational Procrastination Scale (IPS)**

		**Factor 1**	**Factor 2**
**IPS1**	I put things off so long that my well-being or efficiency unnecessarily suffers	.69	
**IPS2**	If there is something I should do, I get to it before attending to lesser tasks (R)		.72
**IPS3**	My life would be better if I did some activities or tasks earlier	.68	
**IPS4**	When I should be doing one thing, I will do another	.55	
**IPS5**	At the end of the day, I know I could have spent the time better	.64	
**IPS6**	I spend my time wisely (R)	.43	.46
**IPS7**	I delay tasks beyond what is reasonable	.64	
**IPS8**	I procrastinate	.64	
**IPS9**	I do everything when I believe it needs to be done (R)		.79

*Note*: Items designated with an (R) are reversed, meaning that a score of 5 instead equals 1. Extraction method: Principal component analysis. Rotation method: Varimax-rotation with Keiser normalization. Coefficients smaller than .40 are suppressed.

#### Factor validity of the PPS and the STS

The PCA of the PPS generated four factors that met the Keiser-criterion of eigenvalue one or higher. However, since the average communality was less than .60, the scree-plot, the number of items per factor, the cross-loadings, and the number of non-trivial factors were examined to determine the number of factors to extract (Zwick & Velicer, 1986). Finally two factors were selected, accounting for 40.92% of the variance. Varimax-rotation was performed and the resulting factor loadings for the PPS are reported in Table 5. These focused on delaying decision making, not meeting deadlines, and missing appointments (factor 1), compared to starting late, lagging behind, and wasting time on other things (factor 2), but not items regarding failure.

**Table 5:** **Rotated component matrix for the two factor solution of the Pure Procrastination Scale (PPS)**

		**Factor 1**	**Factor 2**
**PPS1**	I delay making decisions until it’s too late	.67	
**PPS2**	Even after I make a decision I delay acting upon it	.44	
**PPS3**	I waste a lot of time on trivial matters before getting to the final decisions	.68	
**PPS4**	In preparation for some deadlines, I often waste time by doing other things		.49
**PPS5**	Even jobs that require little else except sitting down and doing them, I find that they seldom get done for days		.72
**PPS6**	I often find myself performing tasks that I had intended to do days before		.45
**PPS7**	I am continually saying “I’ll do it tomorrow”		.57
**PPS8**	I generally delay before starting on work I have to do		.77
**PPS9**	I find myself running out of time	.53	
**PPS10**	I don’t get things done on time	.59	
**PPS11**	I am not very good at meeting deadlines	.64	
**PPS12**	Putting things off till the last minute has cost me money in the past	.46	

*Note*: Extraction method: Principal component analysis. Rotation method: Varimax-rotation with Keiser normalization. Coefficients smaller than .40 are suppressed.

While both of these components seem relevant when assessing clinical levels of procrastination, the first factor does not necessarily fall completely within the current definition of procrastination, i.e., the definition of procrastination does necessitate failure. Repeated failure is on the other hand quite relevant when assessing a clinical procrastinator as it may cause, or moderate, psychological distress, and quality of life. In order to investigate this further, scores from the two components of the PPS were correlated with the MADRS-S, the GAD-7 and the QOLI, as shown in Table 6. These coefficients were twice and almost four times larger for factor 1, the one including failure; *r* = ^±^ .27 to .31, *p* < 0.01, than for component 2, *r* = ^±^ .07 to .15, *p* < 0.01, indicating that this factor may be associated with psychological distress rather than exclusively assessing irrational delay. The correlation with the IPS was also markedly larger for component 2 giving further evidence to the notion that those items deal purely with irrational delay.

**Table 6:** **Correlates between factor scores for the Pure Procrastination Scale (PPS) two factor solution and the other scales**

**PPS**	**GAD-7**	**MADRS-S**	**QOLI**	**IPS**	**STS**
**Factor 1**	0.31	0.28	-0.27	0.35	0.29
**Factor 2**	0.15	0.10	-0.07	0.54	0.33

*Note*: All correlations are significant, *p* < .01. Factor 1 includes failure to meet deadlines and being too late whilst factor 2 seems to strictly deal with irrational delay.

The PCA of the STS generated a single factor with an eigenvalue of at least one, i.e., 4.98, which alone accounted for 45.24% of the variance. This means that the STS seem to measure a single component, that is, susceptibility to temptation.

### Reliability

#### Reliability estimates

Means, standard deviations, reliability estimates, and intercorrelations for all of the included scales are displayed in Table 7. All instruments were significantly correlated, *p* < 0.01. The IPS and the PPS correlated moderately, *r* = .61, *p* < 0.01, or *r* = .79, after correcting for attenuation due to unreliability (presented below within parentheses). Both instruments correlated weakly with the STS, *r* = .32 and *r* = .44, *p* < 0.01. Correlations between the IPS, the PPS, the STS and the other instruments (the MADRS-S, the GAD-7 and the QOLI) indicated some discriminant validity. While all correlations were statistically significant, the corrected coefficients were notably smaller for these scales, ranging from *r* = -.17 (-.21) to *r* = .35 (.42) than within the procrastination battery. The only exception was the STS being nearly as correlated to the IPS as it was to the GAD-7. Furthermore, corrected correlations between the MADRS-S, the GAD-7, and the QOLI were stronger than with the procrastination scales, ranging from *r* = -.40 (-.48) to *r* = .66 (.77), indicating two separate groups of variables.

**Table 7:** **Reliability and correlates among instruments**

		***M***	***SD***	**α**	**1**	**2**	**3**	**4**	**5**
**1**	**IPS**	38.47	3.62	0.76					
**2**	**PPS**	49.26	5.69	0.78	0.61 (0.79)				
**3**	**STS**	42.02	7.07	0.87	0.32 (0.39)	0.44 (0.53)			
**4**	**GAD-7**	8.31	5.26	0.88	0.30 (0.37)	0.35 (0.42)	0.26 (0.30)		
**5**	**MADRS-S**	16.47	7.69	0.83	0.26 (0.33)	0.28 (0.35)	0.17 (0.20)	0.66 (0.77)	
**6**	**QOLI**	0.41	1.73	0.79	-0.19 (-0.25)	-0.25 (0.32)	-0.17 (-0.21)	-0.40 (-0.48)	-0.59 (-0.73)

*Note:* The correlations are reported uncorrected, and in parentheses when corrected for attenuation due to unreliability. All correlations are significant, *p* < 0.01.

#### Reliability and minimal detectable change for the IPS

The IPS had a good internal consistency, Cronbach’s α = .76 (if separated, the procrastination factor had an internal consistency of .72, and the reversed score factor had .53), and all the items in the instrument were judged worthy of retention since there were no items whose absence would raise the alpha-level notably. All items correlated with the full scale at an acceptable level considering the sample size, lowest being *r* = .34.

Correlations between successive weekly measurements on the IPS ranged from .73 to .90, with a median of .84. This was based on the results from the IPS when administered between week two and three, as these two weekly self-report measures had the highest number of valid observations, *n* = 46. The ICC and subsequent analyses were therefore carried out with the data from these weeks. This produced an ICC of .83, indicating excellent reliability, ICC > .75 (Marx et al., 2003). SEM for the IPS was 1.61, and the MDC for the IPS was estimated to 4.47 points. Hence, a change in IPS-score of 4.47 is to be considered a statistically significant difference, i.e., real, *p* < 0.05.

#### Reliability of the PPS and the STS

The PPS and the STS were also shown to have a good internal consistency, Cronbach’s α = for PPS was .78 (if separated, the two factors found in PCA had Cronbach a of .72 and .69). In terms of the STS, Cronbach’s α =. 87, and all of the questions in the scales were deemed worthy of retention. The lowest item-total correlation was .29 for both the PPS and the STS. The correlations between the scales and the internal consistencies for the IPS, the PPS and the STS can be obtained in Table 7.

## Discussion

The PCA for the IPS revealed two factors. The first factor representing general procrastination, accounting for 35% of the variance, and a second factor that simply seemed to reflect the reverse items in the scale. Hence, the IPS seems to be unidimensional, as proposed by Steel (2010), while having an artifact imbedded in the reverse scored items, possibly due to carelessness or satisficing, that is, skimming through the response alternatives in order to preserve cognitive resources (Hinkin, 1995; Schmitt & Stults, 1985; Harvey et al. 1985). Further research is needed in order to assess if the reverse items can be rephrased so that the meaning of the items will be clearer, and to investigate how this can affect the factor structure. The PCA for the PPS generated a two factor solution, explaining approximately 41% of the variance. Both factors seem to be associated with voluntary delay, suggesting there may be a single higher order factor being measured, which is consistent with the conclusions of Steel (2010) and Rebetez et al. (2014). However, the first factor was made up of items dealing not only with delay, but also with failure to meet deadlines and finishing tasks, and was far more correlated with depression, anxiety, and poor quality of life than the second factor. Hence, in a clinical population, the PPS seems to measure procrastination accurately, that is, voluntary delay, but may also consist of a subset of items that measure procrastination-associated failures, and indirectly the impact of procrastination on one’s psychological well-being. Although these findings are preliminary and need to be replicated, Rebetez et al. (2014) found similar evidence for two separate factors, one being related to voluntary delay, and the other being associated with observed delay, that is, the observation of running out of time and not meeting deadlines. The item loadings of the two factors do, however, differ between the current study and that of Rebetez et al. (2014), namely, that items 1-3 load on general procrastination (or voluntary delay) in Rebetez et al. 2014), while these items load on the failure to meet deadlines in the current study (or observed delay). These three items all involve decision-making, originally emanating from the Decisional Procrastination Questionnaire (Mann et al. 1997). Since Steel (2010) found no evidence for the decisional subtype of procrastinators these items may need to be rephrased in light of these inconsistent findings. Furthermore, Rebetez et al. (2014) found a floor effect on item 12 of the PPS, with 80 % of the responses being either 1 or 2. However, the current study found the opposite results, with 66 % of the responses being either 4 or 5. This could indicate that the current study and Rebetez et al. (2014) comprised two distinct populations, and that the PPS picks up different factors accordingly, or, alternatively, that it reflects the removal of the word “money” from the Swedish translation.

In terms of the STS, the PCA revealed only a single factor, accounting for more than 45% of the variance, that is, susceptibility of temptation, and is coherent with the findings of Steel (2010).

The similarities between Steel (2010) and the current study are further confirmed by the correlations between the different scales. The results indicate a high correlation between the IPS and the PPS, *r* = .79, which is at a similar level, *r* = .87, to Steel (2010). This gives further evidence for the unidimensionality of the instruments, and that they can be used interchangeably to share validation efforts. The STS, measuring a different component, correlates to the IPS and the PPS at *r* = .39 to .53, which is comparable to the results obtained by Steel (2010), *r* = .69. The small difference might be explained by the fact that the current study was part of a clinical trial that focused on problems related to procrastination, rather than susceptibility to temptation.

All of the scales yielded good to excellent reliability with Cronbach’s α, ranging from .78 to .87, as well as the ICC for the IPS being .83. In addition, the SEM for the IPS was 1.61 and the MDC 4.47 points, indicating that, in reality, a change of almost five points on the scale is necessary to determine a reliable change in procrastination, which is of particular importance in a clinical context. However, five points may not necessarily indicate a good treatment outcome, and the post treatment results thus need to be considered in light of the baseline severity level.

Furthermore, the correlation matrix showed that the procrastination scales did not correlate highly, *r* = -.17 to -.35 (-.25 to .42 corrected for attenuation due to unreliability), with the other measures of depression, anxiety, and quality of life, suggesting that they do not measure an overlapping construct and are different from each other.

The current study has several limitations that need to be considered when interpreting the results. First, the population recruited for the clinical trial consisted of self-referred participants who perceived themselves to be in need of treatment for procrastination. However, as procrastination is not considered a psychiatric condition, no structured clinical interview could be implemented in order to establish the occurrence and severity of procrastination, for instance, the Structured Clinical Interview for DSM-IV (SCID; First et al., 1996). Hence, the participants may not necessarily have had a clinical problem of procrastination, warranting further research in order to determine whether the self-report measures evaluated in the current study can be used to distinguish a clinical from non-clinical population. Second, although the population was similar to that of Steel (2010) in terms of mean age (38.59 compared to 37.4) and gender (43.4% compared to 57.4% females), the participants may be somewhat older than the average individual experiencing difficulties due to procrastination. According to Steel (2007), problems of procrastination decrease with age, being most prevalent and severe among teenagers and students. The occurrence and severity of procrastination might therefore be more manifest and troublesome for a younger population, which might affect the validity and reliability of the self-report measures used in the current study, and in turn motivate further research. Third, the fact that the participants actively sought treatment could, in itself, be regarded as uncharacteristic of a typical procrastinator, potentially making the population in the current study somewhat different to procrastinators in general. Forth, the instruments used in the current were distributed via the Internet, which might not necessarily correspond to a paper-and-pen administration. However, prior research comparing various self-report measures completed via either the Internet or by paper-and-pen have not found any evidence that the format would affect the responses in a way that would limit their validity or reliability (Luce et al., 2007; Holländare et al., 2010; Grieve & de Groot, 2011; Lindner et al., 2013), indicating that the instruments in the current study might be just as useful when administered by paper-and-pen.

Additional research is warranted in terms of investigating the relationship between various self-report and behavioral measures of procrastination. Preliminary evidence by Krause and Freund (2014) have for instance shown that there might be a difference between assessing procrastination by self-report and behavioral measures, and that self-report measures seem to be more associated with well-being than behavioral measures. In addition, establishing a cut-off to distinguish clinical from non-clinical samples of procrastinators using self-report measures is important, as well as to explore the usefulness of the scales in a clinical context (Klingsieck, 2013). Furthermore, another important issue regarding the different scales is to explore if there are different types of procrastination-related difficulties, which in turn could help tailor the treatment interventions to the specific type. Any future psychometric investigation of the scales should also involve a Confirmatory Factor Analysis in order to further clarify and replicate the findings of Steel (2010), Rebetez et al. (2014), and the current study, in terms of the factor structure of the IPS, PPS and STS.

## Conclusions

The Swedish translation of the scales in the current study seem to measure one general form of procrastination, that is, voluntary delay, as well as susceptibility to temptation, and are deemed both valid and reliable for assessing the occurrence and severity of procrastination via the Internet. The current study supports the use of the scales in a clinical and a non-clinical context in Swedish and similar populations.

## Abbreviations

IPS: Irrational Procrastination Scale
PPS: Pure Procrastination Scale
STS: Susceptibility to Temptation Scale
MADRS-S: Montgomery Åsberg Depression Rating Scale – Self-report version
GAD-7: Generalized Anxiety Disorder Assessment 7-item
QOLI: Quality of Life Inventory
KMO: Keiser-Meyer-Olkin’s test of sampling adequacy
PCA: Principal Component Analysis
ICC: Intraclass Correlation Coefficient
SEM: Standard Error of Measurement
MDC: Minimal Detectable Change
